# Risk Stratification of Dysphagia After Surgical Treatment of Hypopharyngeal Cancer

**DOI:** 10.3389/fsurg.2022.879830

**Published:** 2022-05-19

**Authors:** Hye Ah Joo, Yoon Se Lee, Young Ho Jung, Seung-Ho Choi, Soon Yuhl Nam, Sang Yoon Kim

**Affiliations:** Department of Otorhinolaryngology-Head and Neck Surgery, Asan Medical Center, University of Ulsan College of Medicine, Seoul, Korea

**Keywords:** hypopharyngeal cancer, posterior pharyngeal wall, dysphagia, tracheostomy tube, percutaneous gastrostomy (PEG)

## Abstract

**Objective:**

Hypopharyngeal cancer is managed by either surgical resection or radiation therapy-based treatment. In choosing the treatment modality, the patient’s swallowing function should be considered to achieve optimal treatment outcomes. This study aimed to stratify the risk factors predictive of postoperative dysphagia in hypopharyngeal cancer.

**Study Design:**

Retrospective study.

**Setting:**

Tertiary referral center.

**Methods:**

We enrolled 100 patients who were diagnosed with hypopharyngeal cancer and underwent curative surgery between January 2010 and December 2019, and retrospectively reviewed their medical records.

**Results:**

Postoperative dysphagia occurred in 29 patients (29%) who required a tracheostomy tube or percutaneous gastrostomy tube for feeding or preventing aspiration; additionally, the overall survival rate was lower in those patients than in those without dysphagia. The univariate analysis revealed that postoperative dysphagia was associated with clinical T stage (*p* = 0.016), *N* stage (*p* = 0.002), and surgical resection extent of the larynx and pharynx (*p* < 0.001). Patients who underwent total laryngectomy with total/partial pharyngectomy were more likely to have dysphagia than those in the larynx-preserving pharyngectomy groups (odds ratio [OR] = 3.208, 95% confidence interval [CI] 1.283–8.024, *p* = 0.011). Concerning the posterior pharyngeal wall (PPW), which has an important role in swallowing, patients who underwent resection of ≥1/2 of the PPW were more likely to have dysphagia (OR = 7.467, 95% CI 1.799–30.994, *p* = 0.003).

**Conclusions:**

Surgical resection extent was proportionally associated with dysphagia in hypopharyngeal cancer patients. Patients with smaller lesions but no laryngeal invasion had better postoperative swallowing function than patients with larger lesions or laryngeal involved lesions. Preserving the larynx and hypopharyngeal mucosa (especially the PPW) as much as possible can help preserve postoperative swallowing function.

## Introduction

Hypopharyngeal cancer accounts for approximately 5% of head and neck cancer (HNC) cases and its global incidence is about 0.8 per 100,000 (0.3 in women, 1.4 in men). The most frequently involved subsite is the pyriform sinus (70% of cases), followed by the retrocricoid region (15–20%) and the posterior pharyngeal wall (PPW) (10–15%). Delayed detection in the advanced stages due to submucosal spreading of tumors and frequent locoregional or distant metastasis are associated with worse prognosis in hypopharyngeal cancer (1–3). Treatment options for hypopharyngeal cancer include surgery, radiotherapy, and chemotherapy, alone or combined. Surgical management depends on the affected subsites and lesion extent, such as proximity of the hypopharyngeal cancer to the larynx, which often requires laryngectomy and reconstruction. The oncological outcomes as well as the functional outcomes, including swallowing and phonation, should be considered upon choosing a definitive treatment modality.

Due to invasive growth during destruction of the neighboring critical anatomical structures, the treatment of hypopharyngeal cancer can leave patients with physical, functional, and emotional impairments (4–6). Among these impairments, dysphagia occurs in up to 50% of HNC survivors, frequently among patients with advanced-stage disease (7). Dysphagia induces malnutrition, weight loss, dehydration, aspiration pneumonia and chronic aspiration, all of which are potentially life-threatening. Additionally, dysphagia even leads to social isolation and psychological distress such as anxiety and depression, ultimately deteriorating patient’s quality of life (QOL) (8).

A radiation therapy-based approach (concurrent chemoradiation therapy, [CCRT]) is considered to preserve organs instead of radical resection of hypopharyngeal cancer. Instead of this approach, organ- and function-preservation surgery is an alternative treatment strategy in the management of hypopharyngeal cancer. Conservative surgical treatment for hypopharyngeal cancer has also been used to preserve phonatory and swallowing function. However, conservative surgery does not always guarantee adequate swallowing function postoperatively (9). Therefore, risk factors related to dysphagia in postoperative hypopharyngeal cancer patients should be elucidated before organ preservation modalities are chosen. In addition, total laryngopharyngectomy, which is even reconstructed by free flaps, may limit postoperative swallowing function. This study’s primary objective was to investigate the prevalence of postoperative dysphagia and identify its possible risk factors among hypopharyngeal cancer patients who underwent surgical resection.

## Materials and Methods

### Study Population

**Figure 1:**
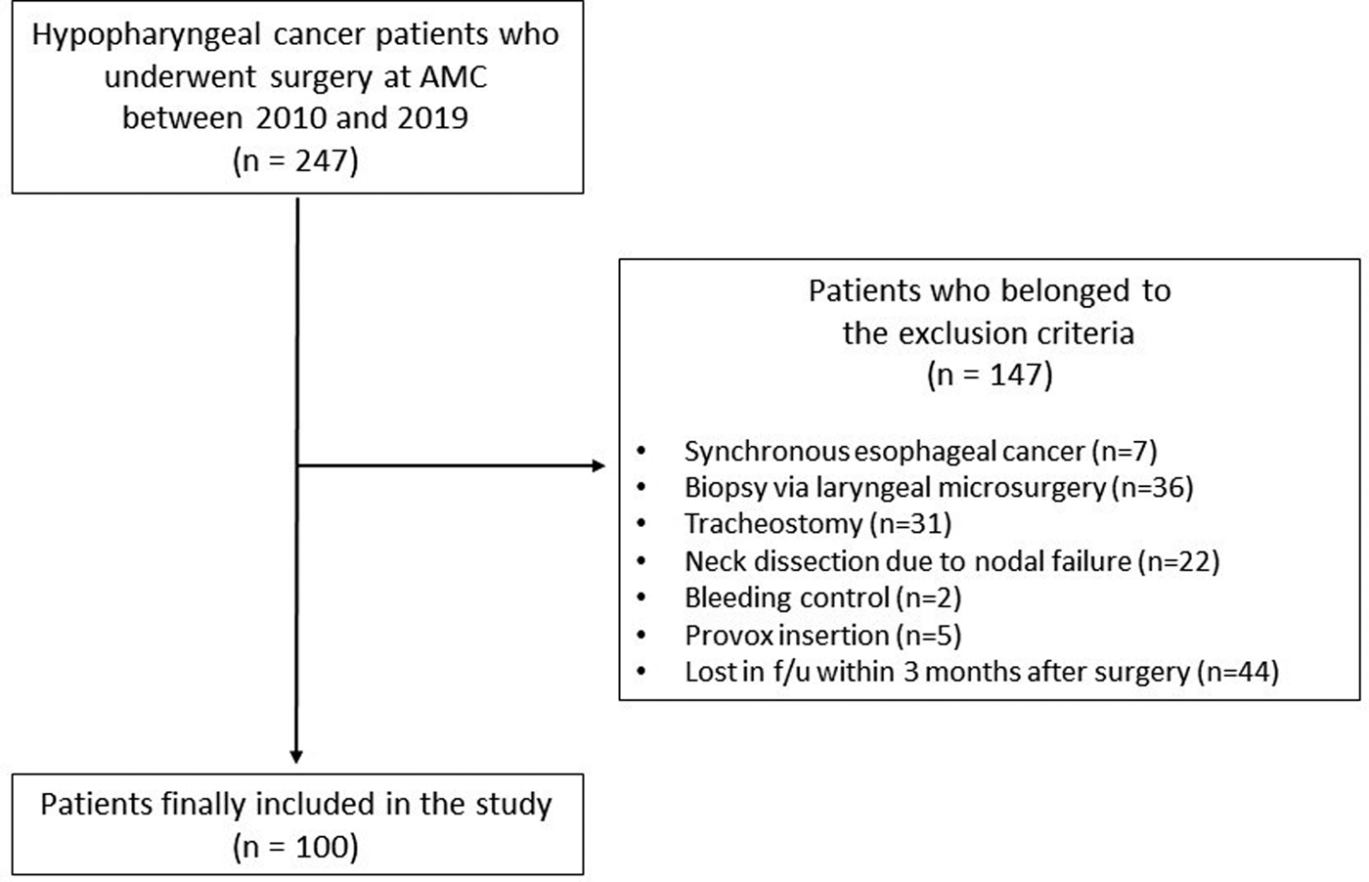
Flowchart of the enrolled patients.

Among patients diagnosed with hypopharyngeal cancer at Asan Medical Center (AMC), those who underwent curative surgery between January 2010 and December 2019 were enrolled in this study and their medical records were retrospectively analyzed. A total of 247 patients with a diagnosis of hypopharyngeal cancer who were ≥18 years of age were identified. Of them, we excluded 147 patients who met the exclusion criteria; ultimately, a total of 100 hypopharyngeal cancer patients treated with curative surgery were included (Figure 1). The exclusion criteria were as follows: (1) a history of previous treatment for upper aerodigestive tract cancer including the larynx, hypopharynx, and cervical esophagus; (2) not having undergone curative surgery, such as biopsy, tracheostomy, or only neck dissection due to nodal failure; and (3) having been lost to follow-up within 3 months postoperatively.

The preservation rate of the swallowing function after surgical treatment was evaluated primarily. Patients with persistent tracheostomy pertaining to aspiration or percutaneous endoscopic gastrostomy (PEG) tube dependency were defined as having severe dysphagia. We evaluated patients in whom a tracheostomy tube or PEG tube was maintained at the last outpatient follow-up visit as having dysphagia.

This study was approved by the institutional review board of AMC (IRB no.: 2021-1071) and performed in accordance with the tenets of the Declaration of Helsinki. The IRB waived the requirement for informed consent considering low risk associated with a retrospective review of data.

### Variables

The preoperative variables included age, sex, neoadjuvant chemotherapy, primary cancer subsite, and cancer stage (TN stage) at diagnosis. Cancer staging was evaluated based on the American Joint Committee on Cancer Staging Manual (AJCC 8^th^ edition) (10). The intra- and postoperative variables included surgical approach, surgical resection extent, PPW resection status, adjuvant treatment, and follow-up period.

For patients who presented with locally advanced hypopharyngeal cancer in whom margin- free resection was considered difficult(mostly those with stage IV diseases), the treatment plans were discussed at AMC’s multidisciplinary cancer clinic. After sufficient consultation between doctors and explanations were made to the patients, the use of neoadjuvant chemotherapy to reduce tumor size and improve surgical resectability was primarily considered. After 2 or 3 cycles of chemotherapy, the treatment response of the primary tumor was assessed by radiologic exams and surgical resection was planned accordingly.

Surgical approaches were classified into four groups: (1) open; (2) transoral laser microsurgery (TLM) using laryngoscope, microscope and CO2 laser; (3) transoral videolaryngoscopic surgery (TOVS) using endoscope; and (4) transoral robotic surgery (TORS).

**Figure 2:**
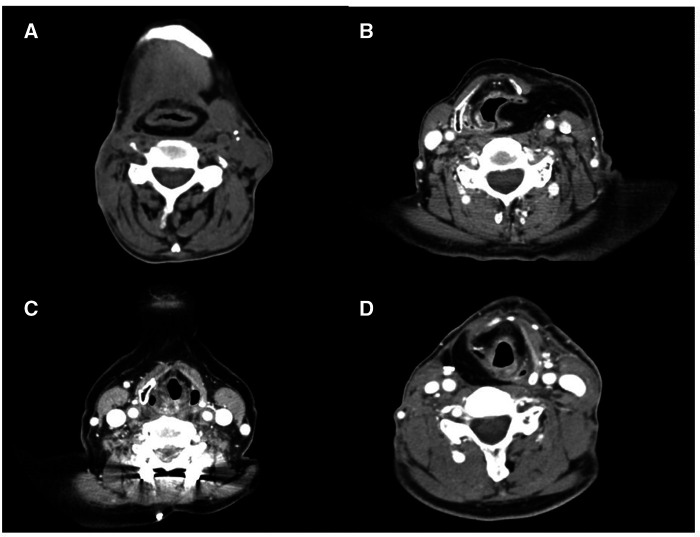
Postoperative neck computed tomography scans of each group classified by surgical resection extent. (**A**) Patient who underwent total laryngopharyngectomy with anterolateral thigh free flap (ALT FF) reconstruction (group 1). (**B**) Patient who underwent left vertical partial laryngectomy (VPL) and left pharyngectomy via lateral pharyngotomy approach with ALT FF reconstruction (group 2). (**C**) Patient who underwent transoral laser excision of the right posterior pharyngeal wall mass (group 3). (**D**) Patient who underwent right partial pharyngectomy via lateral pharyngotomy approach with ALT FF reconstruction (group 4).

Enrolled patients were classified into four groups according to surgical resection extent of the larynx and pharynx, which was included in statistical analysis. Surgical resection extent was evaluated based on both operation records and postoperative computed tomography (CT) scan findings (Figure 2). Group 1 patients underwent total laryngopharyngectomy. Group 2 patients underwent open partial laryngectomy and partial pharyngectomy. Two patients underwent unilateral vertical partial laryngectomy (VPL), while 10 patients underwent partial resection of the involved laryngeal cartilage portion with partial pharyngectomy. Group 3 patients underwent partial pharyngectomy of less than half of hypopharyngeal mucosa, and group 4 patients underwent resection of more than half of hypopharyngeal mucosa. The patients with a pharyngeal mucosa defect which could not be reconstructed by primary closure, underwent reconstruction of the defect by using anterolateral thigh free flap (ALT FF), radial forearm free flap (RFFF), or gastric pull-up.

Figure 2 shows some examples of postoperative CT scans of each group patient classified by surgical resection extent. Figure 2A is a CT scan of the group 1 patient who underwent total laryngopharyngectomy with circumferential ALT FF reconstruction. Figure 2B shows a CT scan of the group 2 patient who underwent left VPL and left pharyngectomy via lateral pharyngotomy approach with ALT FF reconstruction. Figure 2C shows a CT scan of the group 3 patient who underwent transoral laser excision of right PPW mass. Figure 2D shows a CT scan of the group 4 patient who underwent right partial pharyngectomy via lateral pharyngotomy approach with ALT FF reconstruction.

### Statistical Analysis

Continuous variables are presented as mean or median with standard deviation (SD), while discrete variables are presented as frequency and percentage. The statistical analysis was performed using SPSS software version 24.0 (IBM Corp., Armonk, NY, USA). *p* value less than 0.05 was considered statistically significant.

The relevance of univariate variables with the presence of postoperative dysphagia were analyzed using the independent t-test for continuous variables. Categorical variables were analyzed using Pearson’s Chi square test. Subsequently, statistically relevant univariate variables were further analyzed with multivariate analysis using the binomial logistic regression model. Values from the regression model are reported as odds ratios (OR) with 95% confidence intervals (CI). Pearson’s Chi-square test was used with OR and CI to analyze the impact of preservation of the larynx and hypopharyngeal mucosa. Survival analyses were graphically visualized with Kaplan–Meier curves and compared based on the Mantel–Cox log- rank test.

## Results

The patients’ demographic and clinical characteristics are demonstrated in Table 1. The mean follow-up period was 33.6 months (±26.7). Of the enrolled 100 patients, 96 were male (96%), and 4 were female (4%). Among these patients, 29 patients maintained a tracheostomy tube or PEG tube due to postoperative dysphagia (tracheostomy tube; *n* = 4, PEG tube; *n* = 27, both; *n* = 2). The mean age of the patients was 68.0 (±8.0) years. Age (*p* = 0.385), neoadjuvant chemotherapy (*p* = 0.724), primary subsite (*p* = 0.380), histology (squamous cell carcinoma or not) (*p* = 0.374), surgical approach (*p* = 0.112), and adjuvant therapy (*p* = 0.206) were not significantly associated with postoperative dysphagia.

**Table 1:** Patient characteristics and univariate analysis.

Variables	Total	Postoperative dysphagia		*p*-value
		Yes, *n* (%)	No, *n* (%)	
Demographics				
Sample size	100	29 (29%)	71 (71%)	
Sex				0.857^a^
Male	96	28 (29.2%)	68 (70.8%)	
Female	4	1 (25%)	3 (75%)	
Age (year, mean ± SD)	68.0 ± 8.0	69.1 ± 7.3	67.6 ± 8.3	0.385^b^
Neoadjuvant chemotherapy				0.724^a^
Yes	12	4 (33.3%)	8 (66.7%)	
No	88	25 (28.4%)	63 (71.6%)	
Primary subsite				0.380^a^
Pyriform sinus	59	15 (25.4%)	44 (74.6%)	
Retrocricoid region	6	1 (16.7%)	5 (83.3%)	
Posterior wall	35	13 (37.1%)	22 (62.9%)	
Histology				0.374^a^
Squamous	93	28 (30.1%)	65 (69.9%)	
Not squamous	7	1 (14.3%)	6 (85.7%)	
cT stage				0.016^a^
1, 2	63	13 (20.6%)	50 (79.4%)	
3, 4a	37	16 (43.2%)	21 (56.8%)	
Lymph node metastasis				0.007^a^
N (−)	45	7 (15.6%)	38 (84.4%)	
N (+)	55	22 (40%)	33 (60%)	
cN stage				0.002^a^
0, 1	62	11 (17.7%)	51 (82.3%)	
2, 3	38	18 (47.4%)	20 (52.6%)	
Surgical approach				0.112^a^
Open	56	20 (35.7%)	36 (64.3%)	
TLM	36	6 (16.7%)	30 (83.3%)	
TOVS	2	0 (0%)	2 (100%)	
TORS	6	3 (50%)	3 (50%)	
Surgical resection extent				<0.001^a^
Group 1	30	14 (46.7%)	16 (53.3%)	
Group 2	12	5 (41.7%)	7 (58.3%)	
Group 3	45	3 (6.7%)	42 (93.3%)	
Group 4	13	7 (53.8%)	6 (46.2%)	
Adjuvant therapy				0.206^a^
None	40	9 (22.5%)	31 (77.5%)	
RT	34	9 (26.5%)	25 (73.5%)	
CCRT	26	11 (42.3%)	15 (57.7%)	
Follow-up (m, mean ± SD)	33.6 ± 26.7	28.6 ± 26.3	35.6 ± 26.8	0.236^b^

*CCRT, concurrent chemoradiation therapy; SD, standard deviation; TLM, transoral laser microsurgery; TORS, transoral robotic surgery*

^a^ *Pearson’s Chi square test*

^b^ *Independent t-test*

The clinical T stage (*p* = 0.016), N stage (*p* = 0.002) and presence of lymph node metastasis (*p* = 0.007) were significant variables contributing to postoperative dysphagia. Patients with advanced-stage disease showed a higher occurrence of postoperative dysphagia. The distribution of detailed T and N stages is shown in Table 2. To evaluate the predictive value of surgical extent, the patients were classified into 4 groups according to the extent of surgical resection of the larynx and pharynx as described previously. Fourteen of the 30 patients in group 1 (46.7%), 5 of the 12 patients in group 2 (41.7%), 3 of the 45 patients in group 3 (6.7%), and 7 of the 13 patients in group 4 (53.8%) experienced postoperative dysphagia, showing strong statistical significance (*p* < 0.001). The incidence of postoperative dysphagia was the highest in group 4 (53.8%) and the lowest in group 3 (6.7%). Group 4 presented a higher incidence of dysphagia (53.8%) than group 1 (46.7%) and group 2 (41.7%), but the difference was not statistically significant.

**Table 2:** Distribution of clinical T and N classification of patients with hypopharyngeal cancer.

	N stage							Total
T stage	0	1	2a	2b	2c	3a	3b	
1	17	2	0	1	0	0	1	21
2	19	10	1	9	0	1	2	42
3	4	1	0	5	5	0	0	15
4a	5	4	0	7	5	1	0	22
Total	45	17	1	22	10	2	3	100

*Stage I, 17%; Stage II, 19%; Stage III, 17%; Stage IV, 47%.*

In the multivariate analysis, clinical T stage, N stage, and presence of lymph node metastasis were not associated with postoperative dysphagia (Table 3). Less defect in hypopharynx following surgery (group 3) presented a lower risk of dysphagia than any other type of surgery (Pearson’s Chi-square test, OR = 0.078, CI 0.015–0.419, *p* = 0.003). We considered that the group classified by extent of surgical resection was not a categorical variable with an ordinal scale and unlikely to be fully understood by a binominal logistic regression model.

**Table 3:** Multivariate analysis of factors related to postoperative dysphagia.

Variable	OR	95% CI		*p*-value
		Lower	Upper	
cT stage				
1, 2	1			
3, 4a	1.647	0.468	5.799	0.437
Lymph node metastasis				
N (−)	1			
N (+)	1.200	0.254	5.678	0.818
cN stage				
0, 1	1			
2, 3	0.307	0.071	1.334	0.115
Surgical resection extent				
Group 1	1			0.008
Group 2	1.050	0.241	4.563	0.949
Group 3	12.756	2.387	68.175	0.003
Group 4	0.817	0.189	3.538	0.787

*CI, confidence interval; OR, odds ratio.*

*A binomial logistic regression model was used for the analysis.*

To analyze the impact of laryngeal preservation on postoperative dysphagia, we first compared group 1 (total laryngopharyngectomy) with groups 2, 3 and 4 (larynx-preserving pharyngectomy groups). The patients who underwent total laryngectomy with pharyngectomy were more likely to have postoperative dysphagia than those who underwent larynx-preserving pharyngectomy (Pearson’s Chi-square test, OR = 3.208, 95% CI 1.283–8.024, *p* = 0.011). Additionally, to analyze the impact of hypopharyngeal mucosa preservation on postoperative dysphagia, we compared groups 3 and 4. The patients who underwent partial pharyngectomy with more than half of the hypopharyngeal mucosa were more likely to have postoperative dysphagia than those who underwent partial pharyngectomy with less than half of the hypopharyngeal mucosa (Pearson’s Chi-square test, OR = 16.333, 95% CI 3.297–80.924, *p* < 0.001).

In addition, we analyzed the role of PPW resection extent in postoperative dysphagia. Among 100 patients, 47 patients underwent PPW resection (resection of <1/2 of the PPW; *n* = 14, resection of ≥1/2 of the PPW; *n* = 33). The patients who underwent resection of more than half of the PPW (45.5%) were more likely to have postoperative dysphagia than those who underwent resection of less than half of the PPW (21.4%, *p* = 0.003). Especially, among the 30 patients who underwent total laryngopharyngectomy and classified as Group 1, 24 patients underwent circumferential resection of the pharynx and 6 patients underwent resection of the pharynx leaving only a narrow strip of the PPW mucosa. There was no statistically significant intergroup difference in postoperative dysphagia (*p* = 0.855).

**Figure 3:**
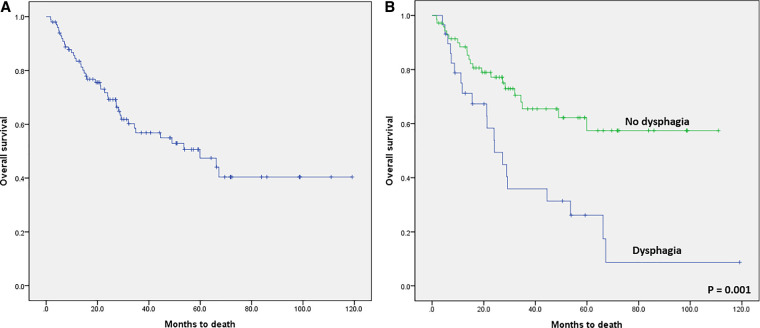
Kaplan–Meier survival curves. (**A**) Overall survival (OS) after curative surgical treatment for hypopharyngeal cancer. The median overall OS was 59.9 months, and the 5-year OS was 47.5%. (**B)** Kaplan-Meier survival curve comparing the patients with dysphagia and patients without dysphagia after curative surgical treatment in hypopharyngeal cancer (log-rank test; *p* = 0.001).

In the survival analysis, the median overall survival (OS) of the 100 enrolled patients was estimated as 59.9 months and the 5-year OS was 47.5% (Figure 3A). The 5-year OS rate was 26.2% in patients with dysphagia and 57.4% in patients without dysphagia (*p* = 0.001, Figure 3B).

## Discussion

In hypopharyngeal cancer, phonation and swallowing functions are important factors in determining the treatment methods along with survival outcome. Radiation-based treatment would be selected as primary treatment to preserve these physiological functions; however, dysphagia may occur even after successful eradication of lesion (6, 11). Surgical resection with or without laryngectomy also affects postoperative swallowing function. In this study, we identified the risk factors predictive of postoperative dysphagia in hypopharyngeal cancer patients who underwent curative surgical resection. The prevalence of dysphagia was 29% in patients who maintained a tracheostomy or PEG tube. The risk factors related to postoperative dysphagia were advanced clinical TN stage and surgical resection extent. Regarding the extent of surgical resection, radical resection of the pharyngeal mucosa seemed to deteriorate swallowing function. Resection of more than half of the PPW increased the risk of postoperative dysphagia; moreover, patients with dysphagia showed a lower survival rate than those without dysphagia.

Dysphagia in HNC is a frequent and overwhelming consequence of the disease and its treatments, especially in the first two years (12). The prevalence of dysphagia after hypopharyngeal cancer treatment has been reported in various studies. Huh et al. retrospectively reviewed the medical records of patients successfully treated for laryngeal and hypopharyngeal cancer with a multimodal treatment approach and reported severe late dysphagia occurrence in 19.2% (19 of 99) of patients (13). They also reported that hypopharyngeal cancer patients are more vulnerable to develop severe late dysphagia than laryngeal cancer patients after non-surgical treatment. Pezdirec et al. evaluated the prevalence of dysphagia in patients treated for HNC in Slovenia using two kinds of questionnaires (6). They reported that 41.3% of HNC patients experienced various swallowing difficulties and especially the prevalence of dysphagia in the hypopharyngeal and laryngeal cancer patients was 39.2%. Murono et al. reviewed the incidence of PEG tube dependence after CCRT for hypopharyngeal cancer. They reported that 16% (12 of 75) showed PEG tube dependence at 6 months after the completion of treatment (11). Tumor subsite and T stage were related to gastrostomy tube dependence in their report. Anatomical organ preservation does not correlate with preserved swallowing function all the time in patients who received CCRT. Thus, surgical resection may be another option from the viewpoint of preservation of swallowing function aside from phonation.

Our study results are valuable because, while most studies broadly reviewed post-treatment dysphagia in HNC patients, we investigated dysphagia after surgical resection in hypopharyngeal cancer in particular. Regarding the surgical resection of hypopharyngeal cancer, partial resection is amenable in T1, T2, and some T3 tumors, with preservation of swallowing function and phonation. If less than 50% of the lateral wall of the pyriform sinus is affected by the defect, direct primary closure is considered. Otherwise, reconstruction is required using the platysma flap, RFFF or ALT FF. In advanced stage tumors (T3, T4), pharyngectomy with total or partial laryngectomy is necessary (1). Tissue defects resulting from the surgical resection itself adversely affects organ function because its integrity is truncated. Furthermore, the sensory impairment of the upper aerodigestive tract mucosa by surgical treatment can affect laryngeal innervation and its function leading to persistent cough and aspiration into the trachea (6). If the defect is reconstructed by free flaps, complications such as fistula or stenosis caused by scar tissue can occur, leading to dysphagia (1). Thus, this study evaluated the impact of surgical resection extent on swallowing.

A notable finding of this study was that patients who underwent total laryngopharyngectomy were much more likely to have postoperative dysphagia than those who underwent larynx-preserving surgery. We initially thought that as the passage for food was separated from the trachea and reconstructed by the circumferential flap, the incidence of dysphagia would be much lower than that after partial pharyngectomy. Stenosis of the neopharyngeal lumen is one cause of dysphagia in patients who underwent total laryngectomy; this complication was reportedly as high as 33% in the surgery group (14) and over 50% in the CCRT group (15). Petersen et al. reported that the cumulative 5-year incidence of neopharyngeal stenosis after total laryngectomy needing dilatation was 22.8% (16). Approximately half of the patients were completely treated with dilatations a few times to relieve the anatomical stricture. Maclean et al. reported that the peak mid-pharyngeal pressures were significantly reduced in laryngectomy patients compared to the controls (17). They found that pharyngeal propulsive contractile forces are impaired after total laryngectomy; additionally, resistance to bolus flow through the pharyngoesophageal segment is increased. Queija et al. suggested the lack of coordination caused by the adaptation of neopharynx-constricting muscles can cause dysphagia after total laryngectomy (18). Anatomical changes such as neopharyngeal stenosis and functional alterations, such as loss of coordinated muscular contraction in the neopharynx, seem to cause dysphagia in total laryngectomy patients.

More specifically, a functional role of PPW was assumed in the subgroup analysis. Patients who underwent radical resection of the pharyngeal mucosa and PPW were more likely to have postoperative dysphagia. Radical resection of the PPW, which left only a narrow strip of PPW mucosa, seemed to be questionable to prevent or ameliorate postoperative dysphagia and swallowing. In the pharyngeal stage of swallowing, pharyngeal shortening and PPW elevation have an important role in making pharyngeal propulsive forces that enable the swallowing of foods (19–21). Functional impairment of PPW induces pharyngeal dilation and reduces pharyngeal contractions. For example, the PPW is thinner and less contractile in elderly populations than in younger populations (22). Murono et al. reported that the PPW was the most significant tumor-related factor for PEG tube dependence after the completion of CCRT for hypopharyngeal cancer (11). This was considered due to the circumferential tumor invading-fields of the PPW being wider than the other subsites. Evangelista et al. reported that the PPW thickness increased significantly after radiation therapy in HNC and that increased PPW thickness was an independent risk factor for increased pharyngeal residue, worse penetration-aspiration scores, and increased post-deglutitive aspiration (19). Based on these reports, PPW impairment induced by radical surgical resection also seems strongly associated with postoperative dysphagia. Therefore, we suggest that the extent of PPW resection should be carefully reviewed preoperatively, and it should be performed conservatively.

This retrospective study has several limitations. First, we believed that the minimal approach would prevent postoperative dysphagia, but surgical approach was not significantly associated with postoperative dysphagia in the statistical analysis. This result was attributed to the small number of patients who underwent endoscopic or robotic surgery. Surgical approaches would depend on clinical T stage rather than location or lesion’s complexity. Second, regarding surgical resection extent, prevalence of postoperative dysphagia was the lowest in group 3 (*n* = 45, 6.7% had dysphagia). The other patients (*n* = 55) had a similar prevalence of dysphagia without a statistically significant difference (group 1, 46.7%; group 2, 41.7%; and group 4, 53.8%). Therefore, in analyzing the impact of preserving larynx in postoperative dysphagia, a comparison between group 1 (total laryngopharyngectomy) and groups 2, 3, and 4 together (larynx-preserving groups) can induce some biased results due to group 3’s good functional outcome and high number of patients. However, we believe that it is still meaningful and necessary to analyze the impacts of preserving the larynx and hypopharyngeal mucosa respectively, by current grouping and statistical analysis. Third, there was a lack of uniformity in the follow-up periods of the enrolled patients and other confounding variables associated with retrospective reviews. We believe that a future prospective cohort study or randomized controlled trial (RCT) based study with a long term follow-up will enable the more systematic reporting of postoperative dysphagia in hypopharyngeal cancer. At last, another important point is that postoperative dysphagia was only evaluated by the maintenance of a tracheostomy or PEG tube at the last outpatient follow-up visit; therefore, the true dysphagia rate could be underestimated. In this study, we could not evaluate subjective dysphagia. Questionnaires, such as the MD Anderson Dysphagia Inventory (MDADI) score (23) and the Eating Assessment Tool 10 (EAT-10) (24), would aid in the evaluation of postoperative swallowing function. Objective clinical test results for dysphagia, such as those obtained on the video fluoroscopic swallowing test (VFSS) and fiberoptic endoscopic evaluation of swallowing (FESS) would enhance the quality of further studies. We believe that tracheostomy or PEG tube dependency are the most important dysphagia-related factors affecting patient’s postoperative QOL. Thus, we used these as the indicators of postoperative dysphagia in this study. We will consider performing a future prospective cohort study or RCT with comprehensive and full evaluations of postoperative dysphagia. Despite the limitations, our findings are noteworthy and may assist the decision-making process of head and neck surgeons for choosing or initiating organ-preserving modalities in hypopharyngeal cancer patients.

## Conclusions

In conclusion, our study findings suggest a 29% prevalence of postoperative dysphagia in hypopharyngeal cancer patients who underwent surgical resection. Surgical resection extent was a significant risk factor of postoperative dysphagia. Patients with smaller lesions and no laryngeal invasion (group 3) had better postoperative swallowing function than patients with larger lesions or laryngeal involved lesions. Preserving the larynx and hypopharyngeal mucosa as much as possible would help preserve swallowing function after surgical resection of hypopharyngeal cancer. Since dysphagia occurred in some patients who underwent resection of the entire pharynx, which was replaced by a free flap, pharyngeal function is critical to postoperative swallowing function. The swallowing function would be maintained by preserving a substantial portion of the PPW, rather than anatomical shape. PPW resection extent in particular should be considered when deciding the treatment options to preserve the swallowing function of hypopharyngeal cancer patients.

## Data Availability

The original contributions presented in the study are included in the article/Supplementary Material, further inquiries can be directed to the corresponding author/s.
